# Prevalence and genetic diversity of rotavirus infection in children with acute gastroenteritis in a hospital setting, Nairobi Kenya in post vaccination era: a cross-sectional study

**DOI:** 10.11604/pamj.2017.26.38.10312

**Published:** 2017-01-24

**Authors:** Mary-Theresa Agutu, Julliette Ongus, Janeth Kombich, Rose Kamenwa, James Nyangao, John Kagira, Adelaide Ayoyi Ogutu, Austine Bitek

**Affiliations:** 1 Institute of Tropical Medicine and Infectious Diseases, Kenya; 2 Jomo Kenyatta University of Agriculture and Technology, Kenya; 3 University of Kabianga, Kenya; 4 Aga Khan University Hospital, Kenya; 5 Kenya Medical Research Institute, Kenya; 6 Animal Health and Industry Training Institute, Kenya; 7 Zoonotic Disease Unit, Kenya

**Keywords:** Rotavirus, prevalence and genotype, vaccines

## Abstract

**Introduction:**

Rotavirus is the leading cause of severe diarrhoea among infants and young children. Each year more than 611 000 children die from rotavirus gastroenteritis, and two million are hospitalized, worldwide. In Kenya, the impact of recent rotavirus vaccinations on morbidities has not been estimated. The study aimed at determining the prevalence and identity of rotavirus strains isolated from rotavirus-associated diarrhoea in vaccinated children presenting with acute gastroenteritis.

**Methods:**

Two hundred and ninety eight specimen from children presented at Gertrude Childrens’ Hospital from January to June 2012 were tested by EIA (Enzyme-linked Immunosorbent Assay) for rotavirus antigens. Molecular characterization was conducted on rotavirus-positive specimens. Extracted viral RNA was separated by polyacrylamide gel electrophoresis (PAGE) and the specific rotavirus VP4 (P-types) and VP7 (G-types) determined.

**Results:**

The prevalence rate of rotavirus was 31.5% (94/298). Of the rotavirus dsRNA, 57 (60.1%) gave visible RNA profiles, 38 (40.4%) assigned long electropherotypes while 19 (20.2%) were short electropherotypes. The strains among the vaccinated were G3P [4], G12P [6], G3P [6], G9P [4], G mixed G9/3P [4] and G1/3P [4]. Specifically, the G genotypes were G9/3 (5.3%), G9 (4.3%), G3 (4.3%), G12 (2.1%) and mixed G1/3 (1.1%). The P genotypes detected were P [4] (5.3%) and P [6] (5.3%).

**Conclusion:**

The present study demonstrates diversity in circulating genotypes with emergence of genotypes G3, G9, G12 and mixed genotypes G9/3 and recommends that vaccines should be formulated with a broad range of strains to include G9 and G12.

## Introduction

Diarrhea is a leading killer of children in Kenya, causing approximately 9 percent of deaths in children less than five years of age [1]. It is estimated that 27% of all under five diarrheal disease hospitalization in Kenya is caused by rotavirus infection [2]. Current global estimates of mortality attributed to rotavirus-associated disease is 611,000 in children less than 5 years old [3] and mortality figures for sub-Saharan Africa total 145,000 annually [4]. Studies in Nigeria [5], Tunisia [6] and Kenya [7] have demonstrated that close to 90% of all children are infected with rotavirus by 2 years of age. In Kenya, the peak age of contracting gastroenteritis in children is 6- 24 months [8–10]. It is estimated that, in Kenya, 68 deaths, 132 hospitalizations, and 21,800 clinic visits per 100,000 children aged less than 5 years annually are attributable to rotavirus diarrhoea [11]. Rotavirus belongs to the family Reoviridae, non-enveloped with a triple-layered icosahedral protein capsid and a genome of 11 double stranded RNA segments [12]. Rotavirus is the most common etiological agent associated with severe gastroenteritis leading to dehydration and death in young infants’ worldwide [3]. Rotaviruses are classified into groups, subgroups, serotypes and on the basis of electrophoretic migration of gene segments. The group and subgroup specificity are present on the inner capsid VP6. Thus, currently only rotavirus groups A, B and C have been identified as human and animal pathogens, while groups D, E, F, and G have only been identified in animals and birds [13–15]. Majority of rotavirus diarhoea infections are caused by group A rotaviruses [16]. Group B rotavirus infection are uncommon but have recently been associated with outbreaks in China and India [17]. Group C rotavirus has been isolated in Kenyan children [18]. The genes encoding the outer capsid VP7 and VP4 form the basis of classification of group A rotaviruses into G and P genotypes, respectively [19]. There are twenty P genotypes with P [4], P [6], and P [8] most frequently associated with human infections. Further, there are 14 G genotypes with G-types 1, 2, 3 and 4, accounting for 80% of human infections. G8 and G9 are emerging as common genotypes with G9 being the most common in Kenya [20]. There are currently two orally administered rotavirus vaccines; Rotarix^®^ (GlaxoSmithKline, Rixensart, Belgium) and Rotateq^®^ (Merck &Co., Whitehouse Station, NJ), which have been available in Kenyan hospitals since 2007. Rotateq^®^ vaccine contains five human-bovine reassortant viruses [W179-9 (G1P [5], SC-2 (G2P [5]), W178-8 (G3P [5]), BrB-9 (G4P [5]) and W179-4 (G6P [8])]. These viruses were generated by crossing the naturally attenuated bovine rotavirus strain WC3 with five unique human rotaviruses each contributing a G1, G2, G3, or G4 VP7 or P[8] VP4 gene to one of the vaccine viruses [21]. Rotarix^®^ vaccine is a human G1P [8] virus RIX4414 which was derived by serial passage in cell culture of a virus recovered from the stool of an infected child [22]. Human rotavirus vaccine has been shown to reduce hospitalizations as a result of gastroenteritis from any cause by up to 42% [22]. The present study was aimed at estimating prevalence of rotavirus gastroenteritis and the genetic diversity of rotavirus among the vaccinated children at the Gertrude’s Children Hospital, Nairobi, Kenya.

## Methods

### Study setting and population

This study was conducted at the Gertrude’s Children’s Hospital (GCH) between January and July 2012. The GCH is a leading Pediatric Hospital in Kenya which offers a variety of healthcare services to infants, school-age children, and teenagers. The laboratory work was undertaken at the rotavirus laboratory in the Centre for Virus Research (CVR) of the Kenya Medical Research Institute (KEMRI), Nairobi, Kenya. The study population comprised children presenting with gastroenteritis and attending the Gertrude’s Children’s Hospital. All children aged less than 5 years presenting with gastroenteritis at the hospital’s outpatient department, or during their first 48 hours of hospitalization and whose parents/guardians consented to the study, were included in this study. Those children who had developed gastroenteritis 48 hours after admission, or those with bloody stool with gastroenteritis and those whose parents/guardians did not give consent, were excluded from the study.

### Ethical clearance

Ethical clearance for this study was obtained from the Scientific and Ethical Review Committees of the Kenya Medical Research Institute (KEMRI). Authorization to conduct the study was obtained from the Gertrude’s Children Hospital (GCH).

### Research instruments

A structured questionnaire was used to collect the patients’ clinical and demographic data, both from the hospital records and from the consenting parents/guardians.

### Study design and sample size determination

This was a hospital based cross-sectional study. The required sample size was calculated using Fischer et al (1998) formula. The sample size was calculated based on a previous prevalence study of rotavirus gastroenteritis in an urban hospital in Nairobi, which had been established to be 28.4% [23]. To detect this with a precision of 5% and confidence level at 95%, at least 313 patients was required. Only 298 had adequate sample for analysis.

### Specimen collection and processing

Stool specimen was collected in a sterile polypot from a patient presenting with symptoms of gastroenteritis. They were stored in a -20°C freezer then transported in cool boxes to the processing laboratory at the Kenya Medical Research Institute (KEMRI), Nairobi. All procedures for working with biosafety level 2 (BSL2) agents such as rotavirus were observed as indicated in the WHO biosafety manual. All biohazardous materials were disposed off in the KEMRI incinerator. One milliliter of 10% (w/v) feacal suspension was prepared in phosphate-buffered saline (PBS), vortexed and clarified by centrifugation. The clarified supernatant was tested for the presence of group A rotavirus antigen using the commercial rotavirus antigen-detection Enzyme Linked Immunosorbent Assay (ELISA) (ProSpectTM Rotavirus Microplate Assay, Oxoid Ltd, United Kingdom). The procedures for detection were undertaken according to the manufacturer’s instructions.

### Molecular analysis of ELISA-positive samples

The ELISA-positive samples were analyzed by polyacrylamide gel electrophoresis (PAGE) to identify the presence of rotavirus double stranded RNA. The procedure was as stipulated in the WHO Manual of Rotavirus detection and characterization methods [24]. The rotavirus dsRNA was run on 10% polyacrylamide resolving gels using a large format gel electrophoresis system (Hoefer SE600) and 3% spacer gel was used to enhance resolution of the ds RNA segments. Thirty microlitres (30µl) of each sample was loaded onto gels and electrophoresis was conducted at 100 V for 16-20h at room temperature. The gels were stained using silver staining to group the rotavirus in electropherotypes as described in in the Laboratory Manual developed by the African Rotavirus Workshop in South Africa, 2002 [25]. Reverse transcriptase/polymerase chain reaction (RT-PCR) amplification was performed on the rotavirus ds RNA as described by the South and West African Regional Rotavirus Laboratories. The extracted ds RNA was re-suspended in 20µl of sterile iodized water and stored for use in PCR reactions. Briefly, the dsRNA was denatured by boiling at 94º C for 5 min followed by chilling in ice. The ds RNA was then reverse transcribed by incubating with reverse transcriptase and deoxynucleotides for 30 min at 42º C. The resultant cDNA was amplified in magnesium-dependent PCR. Briefly, for the G typing, a full-length 1062 gene segment 9, encoding for the VP7 glycoprotein was reverse transcribed and amplified by using primers sBeg9 [nucleotide (nt) 1-21, 5’-GGCTTTAAAAGAGAGAATTTC-3’] and End 9 (nt 1062-1036, 5’ GGTCACATCATACAATTCTAATCTTAAG-3’) followed by genotyping with cocktail of primers specific to six human serotypes G1- G4, G8 and G9 (aBT1, aCT2, aET3, aDT4, aAT8 and AFT9) and the consensus primer RVG 9 as described by [26, 27] shown in Table 1. For VP4 genotyping a full length 876 gene segment 4, encoding for the VP4 was reverse transcribed and amplified by using outer primers Con3 and Con2 as previously described [28] shown in Table 2 followed by genotyping with cocktail of primers specific to the five human P genotype P[4], P[6], P[8], P[9], and P[10] (2T-1, 3T-1, 1T-1, 4T-1, 5T-1) and consensus primer as Gentsch describes. PCR fragments were analyzed on 2% TAE agarose gels at 80-90 volts with appropriate molecular weight marker to determine the genotype of rotavirus strain. PCR bands were compared with molecular weight markers.

**Table 1:** Oligonucleotide primers for G serotyping as designed by Gouvea *et al.,* 1990 and Gault *et al*.; 199

Primer	Sequence (5’-3’)	Position(nt)	Strain (genotype)
SBeg	GGCTTTAAAAGAGAGAATTTC	1-21	Group A
Beg9	GGCTTTAAAAGAGAGAATTTCCGTCTGG	1-28	Group A
End9	GGTCACATCATACAATTCTAATCTAAG	1062-1036	Group A
EndA	ATAGTATAAAATACTTGCCACCA	922-944	Group A
aAT8	GTCACACCATTTGTAAATTCG	178-198	69M (G8)
aBT1	CAAGTACTCAAATCAATGATGG	314-335	Wa (G1)
aCT2	CAATGATATTAACACATTTTCTGTG	411-435	DS-1 (G2)
aDT4	CGTTTCTGGTGAGGAGTTG	480-498	ST-3 (G4)
aET3	CGTTTGAAGAAGTTGCAACAG	689-709	P (G3)
aFT9	CTAGATGTAACTACAACTAC	757-776	WI61 (G9)
RVG9	GGTCACATCATACAATTCT	1062-1044	Group A

**Table 2:** Oligonucleotide primers for P genotype PCR typing as designed by Gentsch et al., 1992

Primer	Sequence (5’-3’)	Position (nt)	Strain (genotype)
1T-1	ACTTGGATAACGTGC	339-356	KU [P8]
2T-1	CTATTGTTAGAGGTTAGAGTC	474-494	RV5 [P4]
3T-1	TGTTGATTAGTTGGATTCAA	259-278	1076 [P6]
4T-1	TGAGACATGCAATTGGAC	385-402	K8 [P9]
5T-1	ATCATAGTTAGTAGTCGG	575-594	69M [P10]
Con3	TGGCTTCGCCATTTTATAGACA	11-32	Group A
Con2	ATTTCGGACCATTTATAACC	868-887	Group A

### Data management and analysis

Data coding and analysis was performed done using the SPSS Version 20.0 software. Pearson’s chi-square was used to determine associations. Level of significance was fixed at 0.05 (p=0.05).

## Results

Three hundred and thirty one (331) participants who met all the inclusion criteria were recruited. Of these, 298 stool specimens were examined in the final analysis. Ninety four (94) (31.5%) specimens were positive for rotavirus (Table 3). Rotavirus infection was most common in children below 2 years of age. Among those infected, males were 46 (48.9%) while the females were 48 (51.1%) and 57 (60.1%) were still breastfeeding. Over 60% of the rotavirus cases had z- scores above median showing that they were of good nutritional status with the majority (41.5%) recording a median score (Table 3). Among the rotavirus positive cases, 33 (35.1%) had been vaccinated using either of the rotavirus vaccines available. Of these, 26 (78.8%) experienced diarrhoea for less than 3 days and only 2 had diarrhea for more than 5 days (Table 4). Only 12 among those who were vaccinated stayed long in the hospital (more than 4 days). All 29 who had some dehydration were treated with intravenous fluids. A total of 94 EIA rotavirus-positive stool samples were subjected to PAGE and visible dsRNA migration patterns were obtained in 57 (60.1%) samples. Long electropherotypes were 38 (0.4%) while 19 (20.2%) displayed short patterns. Reverse transcription was done on 83 samples which had adequate RNA extracted. Overall, the results indicated the most predominant rotavirus strains were G3P [4] (8.5%) followed by G12P [6] (4.3%), G9P [6] (2.1%) and G3P [6] (1.1%). Many strains detected as mixed strains included G9/3P [4] (4.3%), G9/3P [6] (1.1%) and G1/3P [4] (1.1%). Others which were only partially G or P were as follows: G9P [NT] (9.6%), G9/3P [NT] (6.4%), G3P [NT] (3.2%), G1P [NT] (3.2), GNTP [4] (2.1%), and GNTP [6] (2.1%) (Table 5).

**Table 3:** Characteristics, clinical symptoms and treatment of children <5 years of age hospitalized with rotavirus and non-rotavirus gastroenteritis

characteristics	Rotavirus-positive cases	Rotavirus-negative
	n=94(%)	n=204(%)
**Sex**		
Male	46(48.9)	118(57.8)
Female	48(51.1)	89(43.6)
**Age in months**		
0-12	35 (37.2)	101(49.5)
13-24	37(39.4)	59(29)
25-36	6(6.4)	23(11.3)
37-48	8(8.5)	16(7.8)
49-60	8(8.5)	8(3.9)
**Vaccination status**		
Rotarix^®^	21(22.3)	38(18.6)
Rotateq^®^	4 (4.3)	21(10.3)
Vaccine used unascertained	8 (8.5)	30(14.7)
Not vaccinated	61(64.9)	115(56.4)
Breast feeding	57(60.1)	119(58.3)
**Z-scores**		
Median	39 (41.5)	83(40.7)
-1 sd	14(14.9)	46(22.5)
-2 sd	9(9.6)	14(6.9)
-3 sd	1(1.1)	8(3.9)
1 sd	16(17)	33(16.2)
2 sd	4(4.3)	6(2.9)
3 sd	5(5.3	5(2.5)

**Table 4:** Clinical symptoms and treatment of vaccinated and non-vaccinated children infected by rotavirus

Clinical symptom/ treatment	vaccinated	Not vaccinated
	n=33	n=61
**Diarrhoea**		
**Duration of diarrhoea**		
Equal to or < 3 days	26	41
>3<5	5	8
>5 days	2	12
**Frequency of diarrhoea**		
< or equal to 3	25	43
4< or equal to 7	8	18
**Vomiting**		
**Duration of vomiting**		
Equal to or < 3 days	25	49
>3<5	7	9
>5 days	1	3
Frequency		
< or equal to 3	23	38
4< or equal to 7	6	17
>7	4	6
**Hospital stay**		
Short < 3 days	18	33
Long “equal to or > 4 days	12	25
**Dehydration**		
No dehydration	4	3
Some dehydration	29	54
Severe dehydration	0	4
**Treatment**		
Oral rehydration	2	3
Intravenous rehydration	29	57

**Table 5:** Distribution of rotavirus genotypes circulating January 2012 to June 2012 among rotavirus positive and vaccinated children

Strains	Rotavirus positive n=94 No. (%)	Vaccinated n=132	Rotarix n=64	Rotateq n=27
G1P[NT]	3 (3.2)	0	0	0
G3P[4]	8 (8.5)	2	1	1
G3P[6]	1 (1.1)	1	1	0
G3P[NT]	3 (3.2)	1	1	0
G9P[6]	2 (2.1)	1	0	0
G9P[NT]	9 (9.6)	3	1	0
G9/3P[4]	4 (4.3)	2	0	0
G9/3P[6]	1 (1.1)	1	0	1
G9/3P[NT]	6 (6.4)	3	2	1
G1/3P[4]	1 (1.1)	1	1	0
GNTP[4]	2 (2.1)	0	0	0
GNTP[6]	2 (2.1)	1	0	0
GNTP[NT]	29 (30.9)	7	3	2
G12P[6]	4 (4.3)	2	2	0
Not genotyped	18 (19.1)	107	52	22

## Discussion

This study was undertaken to evaluate the diversity of rotavirus (RV) genotypes and prevalence of RV infection in vaccinated children. Rotavirus was detected in 31.5% of the children presenting with gastroenteritis attending GCH in Nairobi, Kenya. The results from this study reveal an overall prevalence of rotavirus infection within the estimated ranges from previous studies. A study conducted from 1991 to 1994 demonstrated a prevalence of 22.5% [23] while a review of rotavirus research in children with diarrhea conducted in Kenya between 1975 and 2005 revealed rotavirus prevalence ranging from 11% to 56.2% in children less than 5 years and 6% in neonates [29]. Another study conducted at the Kenyatta National Teaching Hospital in children revealed a prevalence 59% [30]. A study conducted by Kiulia et al, between 2009 and 2011 demonstrated a prevalence of 37.8% and yet another study carried out in selected hospitals from Kiambu County in Kenya demonstrated a prevalence rate of 36.6 % [31]. After the introduction of rotavirus vaccines in the Kenyan market around 2007 after which this study was conducted 39.4% of the RV infected children were between 13 to 24 months in age followed by those less than 12 months at 37.2%. Consistently, most studies indicate that children less than 2 years are most affected by RV infection. This onset of infection correlates well with the decline of maternally acquired antibodies that disappear around 5 months [32]. In the present study gender difference was not significant. This is in agreement with another study looking at risk factors in pediatric diarrhea [33]. Among the rotavirus positive children, fifty four (57.4%) who were not vaccinated had some dehydration as compared to twenty eight (30%) who were vaccinated. A similar percentage among vaccinated and unvaccinated children were treated by intravenous rehydration. In this study among vaccinated children, G9 genotype was most common genotype at 23.4%. This is in agreement with a recent studies in Kenya where prevalence of 13- 15% have been reported [9, 34, 35]. G9 has been recognized as the most widespread of the emerging genotypes. It was first reported in the United States in the early 1980s [36]. Soon after its detection, it disappeared for more than a decade; then re-emerged in the mid-1990s and has been affecting patients to date. Currently, the genotype comprises 4.1% of global rotavirus infections, and accounts for as high as 70% of rotavirus infections as reported by some studies [37]. Genotype G3 was the second most common genotype demonstrated in this study among the vaccinated children at 4.2%. In Kenya, genotype G3 strains were predominant circulating genotypes in the years 1999 and 2000 [29] but further reports on occurrence of this genotype declined. Studies done elsewhere around the world have reported a high occurrence of G3 genotype. A study in Korea and Ghana found G3 to be the second predominant G genotype at 26.4% and 12.7%, respectively [38, 39]. In Tunisia, a study in 2011 found that, G3 was the second most predominant at 25% [40]. Apart from being the predominant single G genotype, the Tunisian study established that it was also found in mixed infection with G9 and G1 at 17.5%. A study in India earlier mentioned detected 3% and 1.9% of diarrheal cases caused by G3 [41, 42]. The genotype G12 was also demonstrated in this study in both the vaccinated and unvaccinated children in equal percentage (4.3%). A study by Kiulia in 2009 to 2011 in Eastern Kenya [34] showed that G12 was found in 3.1% of the samples. Notably, G12 genotypes have been consistently detected in various countries, including in South Africa. [43, 44]. Since its first identification in the Philippines in 1990 [45], it has been reported worldwide [41]. In Malawi, G12 was the predominant circulating strain [46, 47]. G12 was in association with P [6] genotype and was found in young African children with symptomatic rotavirus infection. In the current study, all the four G12 were in found to be in association with P [6] forming G12P [6] combination. This finding concurs with other early studies that have reported the occurrence of this combination of G12P [6] and G12P [4], isolated for the first time in Bangladesh [48] Genotype G1 as single and combined with G3 was also detected in this study. Genotype G2 was not identified and has not been documented for over 8 years in Kenya as documented by Kiulia et al [34]. The current study did not document any case of G1 genotype among vaccinated children although numerous molecular epidemiological studies have indicated that G1 is the most common circulating G type around the world [37, 49, 50]. Interestingly none of the specimens processed demonstrated genotype P [8]. According to these results genotype G1 and P [8] seems to be controlled well given that both are included in both vaccines available.

## Conclusion

In the current study, prevalence of rotavirus infection remains high in spite of vaccination. Notably, vaccine uptake is still very low. Severity of the rotavirus associated gastroenteritis is reduced with reduced hospital stay. The great diversity and re-emerging rotavirus strains observed in this study especially in vaccinated children raises concerns as to whether the vaccine strains G1P[8], G1,G2,G3 and G4 would evoke sufficient heterotypic protection against other strains not present in the vaccine. This emphasizes the need for long-term rotavirus surveillance in order to establish the effectiveness of vaccines. The findings of high prevalence of G9 and the emergence of G12 strains in Kenya may have implications in the assessment of the efficacy of new rotavirus vaccines. Therefore, we recommend that vaccines should be formulated with a broad range of strains for better protection to include G9 and G12. The limitation of this study was that some of the guardians or parents presenting the children to the hospital did not exactly know the vaccine that was used. Further surveillance on use of vaccines and diversity of strains against vaccine used need to be done.

### What is known about this topic

- Rotavirus is the most common etiological agent associated with severe gastroenteritis leading to dehydration and death in young infants worldwide (Parashar et al., 2006);
- Close to 90% of all children are infected with rotavirus by 2 years in these settings (Nyangao et al., 2010);
- Safe and effective vaccines are needed, especially in poorer countries where most deaths from the disease occur (Parashar et al., 2009).

### What this study adds

- Overall, 94 of the 298 had detectable rotavirus antigen, representing a prevalence rate of 31.5%.
- The study documents the genetic diversity of group A rotaviruses associated with severe acute gastroenteritis in children less than 5 years of age from a hospital where some of the children were already are vaccinated against rotavirus;
- Emerging strains of G12 and G9 were found among these children infected with Rotavirus.
